# A Review of Controversial Issues in the Management of Head and Neck Cancer: A Swiss Multidisciplinary and Multi-Institutional Patterns of Care Study—Part 3 (Medical Oncology)

**DOI:** 10.3389/fonc.2019.01127

**Published:** 2019-10-24

**Authors:** Marco Siano, Pavel Dulguerov, Martina A. Broglie, Guido Henke, Paul Martin Putora, Christian Simon, Daniel Zwahlen, Gerhard F. Huber, Giorgio Ballerini, Lorenza Beffa, Roland Giger, Sacha Rothschild, Sandro V. Negri, Olgun Elicin

**Affiliations:** ^1^Department of Medical Oncology, Cantonal Hospital St. Gallen, St. Gallen, Switzerland; ^2^Department of Medical Oncology, Hôpital Riviera-Chablais, Vevey, Switzerland; ^3^Department of Otorhinolaryngology, Head and Neck Surgery, Geneva University Hospital, Geneva, Switzerland; ^4^Department of Otorhinolaryngology, Head and Neck Surgery, Cantonal Hospital St. Gallen, St. Gallen, Switzerland; ^5^Department of Otorhinolaryngology, Head and Neck Surgery, University Hospital Zurich, Zurich, Switzerland; ^6^Department of Radiation Oncology, Cantonal Hospital St. Gallen, St. Gallen, Switzerland; ^7^Department of Radiation Oncology, Inselspital, Bern University Hospital, University of Bern, Bern, Switzerland; ^8^Department of Otorhinolaryngology, Head and Neck Surgery, University Hospital of Lausanne, Lausanne, Switzerland; ^9^Department of Radiation Oncology, Cantonal Hospital Graubünden, Chur, Switzerland; ^10^Department of Radiation Oncology, Cantonal Hospital of Winterthur, Winterthur, Switzerland; ^11^Department of Radiation Oncology, Clinica Luganese SA, Lugano, Switzerland; ^12^Department of Radiation Oncology, Cantonal Hospital Lucerne, Lucerne, Switzerland; ^13^Department of Otorhinolaryngology, Head and Neck Surgery, Inselspital, Bern University Hospital, Bern, Switzerland; ^14^Department of Medical Oncology, University Hospital of Basel, Basel, Switzerland; ^15^Department of Otorhinolaryngology, Lindenhofspital, Bern, Switzerland

**Keywords:** consensus, head and neck cancer, patterns of care, practice patterns, survey

## Abstract

**Background:** The Head and Neck Cancer Working Group of Swiss Group for Clinical Cancer Research (SAKK) has investigated the level of consensus (LOC) and discrepancy in everyday practice of diagnosis and treatment in head and neck cancer.

**Materials and Methods:** An online survey was iteratively generated with 10 Swiss university and teaching hospitals. LOC below 50% was defined as no agreement, while higher LOC were arbitrarily categorized as low (51–74%), moderate (75–84%), and high (≥85%).

**Results:** Any LOC was achieved in 62% of topics (*n* = 60). High, moderate, and low LOC were found in 18, 20, and 23%, respectively. Regarding Head and Neck Surgery, Radiation Oncology, Medical Oncology, and biomarkers, LOC was achieved in 50, 57, 83, and 43%, respectively.

**Conclusions:** Consensus on clinical topics is rather low for surgeons and radiation oncologists. The questions discussed might highlight discrepancies, stimulate standardization of practice, and prioritize topics for future clinical research.

## Introduction

This is the third part of the article “A Review of Controversial Issues in the Management of Head and Neck Cancer: A Swiss Multidisciplinary and Multi-Institutional Patterns of Care Study,” providing the results for the items concerning medical oncology discipline, each followed by a short discussion if deemed relevant. The details of the methodology is presented in the first part of this series.

## Results and Discussion

### Medical Oncology

This section contains some overlapping topics with the previous sections regarding concurrent CRT and induction chemotherapy. The focus remains on the medical oncologists' point of view.

#### Concurrent Chemoradiotherapy

➢ *Cetuximab is preferred in combination with definitive radiotherapy in loco-regionally advanced HNSCC for cisplatin-ineligible patients: moderate LOC (80%)*.

An important question remains which approach is preferred in cases where cisplatin cannot be applied due to contraindications or patient related factors precluding its application (age, performance status, hearing loss etc.). For this situation, cetuximab (1) as alternative choice is favored in 8/10 centers. One center prefers carboplatin, whereas in another center a combination regimen with 5-fluorouracil (5-FU) and mitomycin C (2, 3) vs. Cetuximab is discussed on patient basis.

Different systemic modalities for concurrent treatment were investigated during the last decades. Cisplatin given every 3 weeks remains the standard of care (4, 5). A minimal dose of ≥200 mg/m^2^ cisplatin has to be administered to achieve optimal outcome (6). Nevertheless, only 61% of patients tolerate the standard dose of 100 mg/m^2^ times three (7). Therefore, different alternatives are investigated. Among them, the well-tolerated platinum alternative carboplatin, alone, or in combination with 5-FU was the combination used by the GORTEC group (8). Cetuximab, based on high level evidence (1), was the preferred choice within our survey, despite the lack of randomized comparison to cisplatin at the time of the survey. Recently, two phase III randomized trials showed that cetuximab is associated with inferior overall survival compared to cisplatin even in the low and intermediate risk HPV-associated OPSCC (9, 10). For mitomycin C in combination with 5-FU, one randomized trial showed superiority of CRT in terms of locoregional control and survival to a dose escalated hyperfractionated accelerated radiation therapy schedule without systemic therapy (11, 12). For mitomycin C, as monotherapy or in combination, no randomized phase III data is available, in comparison to standard of care cisplatin or cetuximab.

➢ *No agreement in the radiosensitizer indication in post-operative setting for cisplatin-ineligible patients: no consensus*.

The same question in the adjuvant CRT setting yielded a different pattern: cetuximab was the preferred choice in 4, carboplatin in 5 centers, In the remaining center, the radiation oncologist would prefer 5-fluorouracil with mitomycin c, whereas the medical oncologist would opt for cetuximab, or carboplatin instead.

In the adjuvant setting, no high-level evidence is available for cetuximab. Despite this fact, almost half the centers adopt the data from non-operated locally advanced disease (1) and prescribe cetuximab. Carboplatin is the preferred agent as monotherapy. For mitomycin C as monotherapy or in combination with dicumarol, an improvement was shown but not regarding overall survival (13). For the combination of 5-FU an extrapolation from the existing data from non-operated locally advanced disease is assumed.

➢ *The cisplatin regimen in terms of dose and cycle frequency concomitant with radiotherapy is quite heterogeneous: no consensus*.

Platinum-based regimens are administered weekly in 4/10, every 3 weeks in 5 centers, and every 3 weeks but distributed over 5 days every 3 weeks in 1 center.

Shortly after our survey was completed, data presented at the annual congress of clinical oncology ASCO 2017 was presented and later on published, showing superiority of the 3-weeks application of cisplatin vs. a weekly application (14). Probably, from the four centers applying cisplatin weekly, some would consider changing their opinion.

➢ *All centers prefer to continue the treatment with another systemic agent in patients who cannot complete the planned number of cycles of cisplatin: high LOC (100%)*.

If a patient was not able to continue with cisplatin after ≥1 cycle, systemic treatment is switched to another regimen in 10/10 centers. In one center, treatment is switched to 5-FU and mitomycin c or carboplatin alone. All other centers prefer cetuximab or carboplatin.

We are not aware of any solid data confirming the benefit of any switch strategy, and with which combination, if there is any value at all. Of note, one of the participating centers recently published a hypothesis-generating retrospective study indicating a higher incidence in second primary cancers, when cetuximab was administered after the discontinuation of platinum-based chemotherapy, compared to pure cetuximab, or platinum-based therapy (15).

➢ *Age is not considered as a strict factor regarding the decision whether to administer concomitant chemotherapy: high LOC (100%)*.

There was total consensus (10/10) about administering chemotherapy concomitant with radiotherapy to selected, medically fit patients even older than 70 years.

Even if there is no randomized prospective data confirming the efficacy of a concomitant strategy in this patient group, all centers apply the same regimen as in their younger counterparts. Some analyses show similar outcomes for these patients despite the higher age (16). Biological age seems to be of importance more than chronological age.

➢ *ECE is a well-established high-risk factor for post-operative concomitant CRT indication: high LOC (100%)*.
➢ *In most centers, positive resection margin is considered a high-risk factor for post-operative concomitant CRT indication: high LOC (90%)*.

Risk factors warranting adjuvant concomitant chemotherapy to radiotherapy vary between centers and are elucidated in Table 1.

**Table 1:** Depending on the following risk factors the centers administer concurrent chemotherapy together with adjuvant radiotherapy.

**Center**	**1**	**2**	**3**	**4**	**5**	**6**	**7**	**8**	**9**	**10**
Number of pos. lymph nodes		X	X		X		X		X	
Extracapsular spread	X	X	X	X	X	X	X	X	X	X
Vascular embolism		X								
Perineural disease		X			X					
Positive resection margins	X	X		X	X	X	X	X	X	X
Stage III-IVB disease		X								

#### Induction Chemotherapy

➢ *The use of induction chemotherapy is not part of the routine: low LOC (60%)*.

The use of induction chemotherapy with the intention of increasing oncological outcome is used in 4/10 centers. The other centers either never administer induction chemotherapy, or only do so in rare cases in presence of bulky disease, in which performing an up-front curative CRT with full-dose is not realistically applicable or feasible. An exact specification of the induction regimen was not pointed out [classic TPF regimen (docetaxel, cisplatin, 5-fluorouracil) (17, 18), adapted TPF, other combination chemotherapy].

Induction chemotherapy is a controversial topic in HNSCC. Nevertheless, during the last decade one regimen, applied “classically” or “adapted” showed level I evidence for having better survival compared to radiotherapy alone in selected patients (17, 18). With the standard of care approach of concurrent radiotherapy and cisplatin, trials comparing these two approaches were eagerly awaited. From five randomized phase III trials, only two compared standard concurrent treatment vs. induction with TPF followed by the same treatment (19, 20). All the other trials were underpowered or did not reach their recruitment goal. Moreover, inadequate systemic agents were applied concurrently to radiotherapy. The trial by Hitt et al. showed a trend toward an improvement of overall survival, but was formally negative (19). A trial with an “adapted” TPF regimen also called “Italian” TPF was able to show a marked and impressive overall survival benefit of more than 20 months (20). The trial is controversial for its design, but the main question, whether an induction approach irrespective of the following concurrent treatment (cisplatin and 5-FU or cetuximab), defined after a second randomization, improved outcome was clearly answered. Concerns about a lower rate of completion of radiotherapy and a higher mortality rate were raised, but could in part be refuted by recent trials. Despite these arguments, induction chemotherapy reduces distant metastases rates more prominently than concurrent CRT alone (21). In the particular case of locally advanced laryngeal cancer, value of induction chemotherapy is higher, due to available data and long-term outcome of pivotal trials, showing better outcome with higher larynx-preservation rate (22–24).

Whether to administer induction chemotherapy in nasopharynx cancer or not is an ongoing discussion. The most recently published study by Sun et al. (25) is a well-designed and conducted study, whose results indicate a favorable progression-free survival with the addition of TPF administered before CRT. However, it is important to note the eligibility criteria and the patient collective of this study. Only cN+ patients younger than 60 years old were allowed. Moreover, the distribution of WHO histological subtypes are neither reported nor mentioned in the published article. Considering the dramatic geographic differences of the histology, a direct implementation of the results of a study from China to European and American patients, especially those with non-EBV tumors, is questionable. Nevertheless, for those who find the study results convincing enough to change their practice, the investigators of the same study created a helpful nomogram based on the trial database to predict the extent of potential gain via induction chemotherapy for a given patient (26).

➢ *The use of induction/neoadjuvant chemotherapy for optimal decision-making in locally advanced laryngeal cancer is preferred: low LOC (70%)*.

However, 7/10 centers favor the use of induction/neoadjuvant (the term “neoadjuvant” is rather used, if a surgery is planned afterwards) chemotherapy for decision making purposes concerning larynx preservation (22, 27).

#### Nasopharyngeal, Nasal, and Paranasal Sinus Tumors

➢ *Administration of chemotherapy before the primary treatment of sino-nasal tumors is preferred due to various reasons: low LOC (60%)*.

For the treatment of clinically aggressive, highly proliferating nasal cavity and paranasal sinus tumors, induction/neoadjuvant chemotherapy is considered in 6/10 centers, especially in case of bulky tumors, and/or presence of symptoms to avoid disease progression until start of radiotherapy (5/6), further to achieve clear surgical margins (1/6).

Due to the relatively low incidence and variety of histological subtypes of nasal cavity and paranasal sinus tumors, there is no convincing level of evidence for or against the use of chemotherapy before, during, or after the primary treatment. Nevertheless, it is interesting to see a low but presence LOC among participating centers.

➢ *Concomitant CRT is preferred for the treatment of sino-nasal tumors: moderate LOC (70%)*.

For the treatment of loco-regionally advanced nasal and paranasal sinus tumors, concurrent chemotherapy is regularly administered in 7/10 centers. In 2 centers, it is administered only in selected cases based on tumor board discussion. One center never performs radiotherapy with concomitant chemotherapy.

There is moderate consensus, that locally advanced disease needs multimodality treatment. This according to almost all guidelines available (NCCN, ESMO, etc.). One center seems to diverge from this approach, probably due to toxicity concerns.

➢ *Concerning the indication of adjuvant chemotherapy for nasopharynx cancer, no standard approach was observed: no consensus*.

Among participating centers, adjuvant chemotherapy for nasopharynx cancer is omitted in three out of ten centers; performed in all cases in three centers; in selected cases at four centers. However, when asked, the definition of “selected cases” was not further specified in three centers. In one center selection was based on treatment response and EBV titer if applicable.

Treatment of nasopharyngeal cancer is a field of controversy. Stages > I need multimodality treatment, where CRT is established as the standard of care (28, 29). Further adjuvant chemotherapy, traditionally proposed for years is based on a pivotal Intergroup 0099 study (30), which had its caveats, raising concerns about the quality of the radiotherapy in the trial and highlighting the importance of patient selection. Despite the co-existence of negative trials showing the futility of adjuvant chemotherapy after radiotherapy alone (31, 32) or CRT (33, 34), an added benefit of adjuvant treatment was confirmed by meta-analyses, one published in 2015 of 19 trials with a total of 4,806 patients, showing the most favorable overall survival (HR 0.65; 95% CI, 0.56–0.76) compared to CRT without adjuvant chemotherapy (HR, 0.80; 95% CI, 0.70–0.93) (35). The other meta-analysis including 20 trials and 5,144 patients, showed that the addition of adjuvant chemotherapy to CRT was associated with better PFS compared to CRT only (HR 0.81; 95% CI, 0.66–0.98) (36). On the other hand, the most recently published phase III trial showed no benefit of adjuvant chemotherapy when added to CRT, even though the study only included high-risk patients with detectable post-CRT plasma EBV DNA (37). Moreover, a majority of patients do not tolerate full adjuvant treatment. Therefore, induction treatment was studied within phase III trials and showed differing results. Nevertheless, two phase 3 trials (25, 38) and a meta-analysis (36) were positive for the primary endpoint overall survival.

##### Supportive Measures and Oligometastatic Disease

➢ *Prophylactic use of colony stimulating factors is not preferred during CRT: moderate LOC (80%)*.

In 2/10 centers, prophylactic use of colony-stimulating factors during CRT was reported.

Cautious application of colony-stimulating factors is probably due to reports finding adverse outcome during chemo-radiation (39) and pre-clinical data suggesting tumor proliferation (40) with such agents. Additionally, the efforts of reducing treatment-related mucositis were futile (41, 42). Although not belonging to the same category of agents, it is also worth to note that the use of erythropoiesis-stimulating agents to overcome anemia and hypoxia was shown to cause an unexpected negative outcome (43).

➢ *Induction/neoadjuvant chemotherapy for subsequent decision-making is preferred in oligometastatic HNSCC: low LOC (60%)*.

For the treatment of oligometastatic (defined as up to 3 metastases) cases at the initial diagnosis, 6/10 centers consider administering induction/neoadjuvant chemotherapy, and decide thereafter based on response the final treatment concept (curative vs. palliative). Three centers never pursue this strategy. One center directly treats the locoregional and distant disease with curative intent.

Compared to other tumor entities (e.g., breast, colorectal, prostate, non-small lung cancer, malignant melanoma), the concept of oligometastatic disease and its treatment in HNSCC were not extensively investigated. Retrospective series demonstrate 5-years survival rates of 20% and higher after local ablation by means of surgery or SBRT of oligometastatic disease (44, 45). However, a high level of evidence is still lacking. Moreover, the optimal strategy for the synchronous presentation of the oligometastases at the time of initial diagnosis poses a more specific question, which still remains unanswered. The heterogeneity in the patterns of treatment among our 10 centers seems to reflect this ambiguity.

##### Systemic Treatments for Recurrent/Metastatic Disease

➢ *In first line, EXTREME is the preferred systemic treatment regimen for recurrent/metastatic disease (R/M): low LOC (60%)*.

➢ *The use of 2nd line anti-PD1 checkpoint inhibitors are preferred in anti-EGFR pre-treated and not pre-treated R/M: moderate LOC (70–80%, respectively)*.
➢ *Anti-EGFR pre-treated patients would be encouraged to participate in clinical trials for* ≥*2nd line treatment: low LOC (60%)*.
➢ *Anti-EGFR-naïve patients are considered for anti-EGFR treatment as* ≥*2nd line: low LOC (60%)*.

The EXTREME regimen containing a platinum compound with 5-fluorouracil and cetuximab is considered for patients with R/M and an ECOG performance status 0–2 in 6/10 centers. The remaining four centers do not necessarily consider systemic treatment according to the pivotal EXTREME trial especially for patients with higher ECOG performance status (46). Second-line systemic treatment choice was mostly based on whether or not previous treatment contained cetuximab (Table 2). There was a moderate LOC (70–80%) among the centers about the application of nivolumab in this setting (47). Nevertheless, the general heterogeneity in the R/M setting among participating centers is not to be overlooked.

**Table 2:** Preferred second-line systemic treatments depending on previous anti-EGFR application.

**Center**	**1**	**2**	**3**	**4**	**5**	**6**	**7**	**8**	**9**	**10**
**ANTI-EGFR PRE-TREATED**										
Methotrexate			X				X		X	
Cetuximab							X			
Taxane			C			M			M	
Anti-PD1 antibody^*^	X		X	X	X		X		X	X
Clinical trial		X	X		X		X	X	X	
Best supportive care					X		X		X	
**ANTI-EGFR-NAÏVE**										
Methotrexate			X						X	
Anti-EGFR antibody		X	X		X		X	X	X	
Taxane			C						M	
Anti-PD1 antibody^*^	X		X	X	X	X	X		X	X
Clinical trial			X	X	X				X	
Best supportive care					X					

* *We reassessed second-line treatment choice after approval of novel anti-PD-1 checkpoint inhibitors. These agents were given under the category “compassionate use.” C, combination; M, monotherapy*.

## Conclusion

The findings of our survey indicate a low LOC among head and neck oncologists working in academic and multidisciplinary setting in 10 Swiss institutions. Regarding the results and the discussion concerning the specialties other than medical oncology, the reader is advised to read the corresponding parts of this article. The highest LOC was achieved among medical oncologists, whereas the lowest was observed among head and neck surgeons. On the other hand, this level of disagreement may also depend on the topics chosen for the survey, and not necessarily the heterogeneity within the disciplines. It is also interesting to witness a low LOC regarding topics, where a high level of evidence actually does exist, and vice versa. This article is expected to serve the head and neck oncologists to be aware of their discrepancies and to stimulate discussion toward standardization of practice and prioritize topics of future clinical research.

## Data Availability Statement

All datasets generated for this study are included in the manuscript/supplementary files.

## Author Contributions

GH, MB, OE, PD, and PP: conception and design. OE and PP: collection of data. All co-authors: generation of the initial and final versions of the questions, drafting of the manuscript, and approval of the final version.

### Conflict of Interest

The authors declare that the research was conducted in the absence of any commercial or financial relationships that could be construed as a potential conflict of interest.
